# Sport and exercise medicine and the Olympic health legacy

**DOI:** 10.1186/1741-7015-10-74

**Published:** 2012-07-19

**Authors:** Garry A Tew, Robert J Copeland, Simon H Till

**Affiliations:** 1 Centre for Sport and Exercise Science, Sheffield Hallam University, Collegiate Hall, Collegiate Crescent, Sheffield, UK, S10 2BP; 2 Sheffield Teaching Hospitals NHS Foundation Trust, Royal Hallamshire Hospital, Glossop Road, Sheffield, UK, S10 2JF

**Keywords:** physical activity, chronic disease, primary prevention, rehabilitation, Olympic legacy

## Abstract

London 2012 is the first Olympic and Paralympic Games to explicitly try and develop socioeconomic legacies for which success indicators are specified - the highest profile of which was to deliver a health legacy by getting two million more people more active by 2012. This editorial highlights how specialists in Sport and Exercise Medicine can contribute towards increasing physical activity participation in the UK, as well as how the National Centre for Sport and Exercise Medicine might be a useful vehicle for delivering an Olympic health legacy. Key challenges are also discussed such as acquisition of funding to support new physical activity initiatives, appropriate allocation of resources, and how to assess the impact of legacy initiatives.

## Background

"The success of the Olympic Games depends in no small measure on the legacy it leaves the world" [1].

Event legacy has become an increasingly important aspect of hosting the Olympic Games since its revival in 1896. Early impacts of the Games were typically assessed through changes in sporting or local infrastructure. Nowadays, the focus is much broader, including themes such as culture, economy, environment, image, nostalgia and health. A key feature of the successful London 2012 bid was the commitment to creating a lasting health legacy through a large-scale and sustained increase in sport and physical activity participation [2].

The importance of physical activity in preventing and treating many diseases and conditions is indisputable, as documented in the current physical activity guidelines for the United Kingdom [3]. Unfortunately, modern physical and social environments discourage regular physical activity, with only 39% of men and 29% of women in the UK meeting minimum physical activity recommendations when measured subjectively and about 5% when measured objectively [4]. Countering these influences will require a coordinated approach involving multiple societal, institutional, and departmental collaborations. Specialists in Sport and Exercise Medicine (SEM) can help [5].

The establishment of SEM as a new medical specialty in 2005 was an important step in the Olympic health legacy commitment, along with the development of the National Centre for Sport and Exercise Medicine (NCSEM). This article describes how SEM Specialists and the NCSEM will work towards increasing physical activity participation in the UK, as well as some of the challenges that might be faced.

### How can Sport and Exercise Medicine Specialists help?

Specialists in SEM are trained in education, physical activity and chronic disease, exercise physiology, public health, general practice and musculoskeletal medicine, and are therefore well equipped to lead initiatives focussed on increasing physical activity participation. Ways in which they can do this have recently been proposed [5], including:

i. Providing education to primary and secondary care teams so that exercise prescription is prioritised within the patient's healthcare experience and consistent, evidence-based, effective physical activity advice is provided.

ii. Establishing multidisciplinary teams to provide a single point of referral for patients identified as requiring specialist help, e.g. those with complex medical problems and those requiring specialist help to effect behavioural change.

iii. Working with the fitness industry to maximise accessibility of supported exercise to all patients irrespective of age, co-morbidity, social and cultural position.

iv. Providing a specialist service including clinical exercise testing and risk assessment for those with exercise intolerance, those with co-morbidity, those with chronic pain and pre-operative patients to assess anaesthetic risk.

v. Providing advice on effective physical activity interventions and injury prevention and treatment in the workplace.

### The National Centre for Sport and Exercise Medicine

The NCSEM was launched in January 2012 as part of the health legacy of the London Games. It has a remit to ensure SEM works towards the benefit of health and wellbeing. The NCSEM comprises three partners: Sheffield, London, and the East Midlands. The Department of Health has pledged £30 million to support the development of a capital infrastructure nationally, with £10 million going to each partner. This funding will support the co-location of SEM specialists, allied health practitioners, researchers and patients to enhance the delivery of SEM across the UK.

The specific themes of work will likely differ between the three centres. In Sheffield, the NCSEM is providing a vehicle for key stakeholders to come together to create a legacy programme to establish the city as 'The City of Physical Activity' over the next 20 years and to inform the participation legacy of future Olympic Games. Sponsorship investment in evidence-based initiatives and 'ground-breaking' programmes is currently being sought (July 2012). A comprehensive description of the work programme is beyond the scope of this editorial; however, Table 1 describes seven initiatives that might be considered the "best investments in physical activity" [6].

**Table 1:** Examples of evidence-based physical activity initiatives included in the Sheffield NCSEM work programme (based on [6]).

Initiative	Brief description
1. 'Whole-of-school' progammes	Involves prioritising: regular, highly-active physical education classes; providing suitable environments and resources to support structured and unstructured physical activity throughout the day; supporting walk/cycle-to-school programmes and enabling all of these actions through supportive school policy and engaging staff, students, parents and the wider community.

2. 'Active transport' policies and systems	Increasing 'active transport' through the development and implementation of policies influencing land use and access to footpaths, bikeways and public transport, in combination with effective promotional programmes to encourage and support walking, cycling and use of public transport for travel purposes.

3. Transforming urban environments	Urban planning and design regulations should require mixed-use zoning that places shops, services, and jobs near homes, as well as highly connected street networks that make it easy for people to walk and cycle to destinations. Access to public open space and green areas with appropriate recreation facilities for all age groups are needed to support active recreation. Complete networks of footpaths, bikeways, and public transit support both active travel and active recreation.

4. Physical activity and non-communicable disease (NCD) prevention integrated into primary health care systems	Health care systems should include physical activity as an explicit element of regular behavioural risk factor screening for NCD prevention, patient education and referral. Positive messages about physical activity are important for primary and secondary prevention. Opportunities for NCD prevention should be integrated with communicable disease management systems, tailored to the context and resources available. The focus should be on practical brief advice and links to community-based support for behaviour change.

5. Public education, including mass media to raise awareness and change social norms on physical activity	Both paid and non-paid forms of media can be used to raise awareness, increase knowledge, shift community norms and values and motivate the population to be more active.

6. Community-wide programmes that mobilise and integrate community engagement and resources	Using key settings, such as cities, local governments, schools and workplaces provides the opportunity to integrate policies, programmes and public education aimed at encouraging physical activity. Whole-of-community approaches where people live, work and recreate have the opportunity to mobilise large numbers of people.

7. 'Sport for all' programmes	Building on the universal appeal of sport, a comprehensive sport system should be implemented that includes the adaption of sports to provide a range of activities to match the interests of men and women, girls and boys of all ages, in addition to well-coordinated coaching and training opportunities. However, providing enjoyable physical activity needs to be an explicit priority of sports programmes.

NCD, non-communicable disease; NCSEM, National Centre for Sport and Exercise Medicine.

### Challenges

There is an assumption when using the term 'Olympic legacy' that there will be a positive outcome, which is not always the case. In the context of the London Olympic health legacy, it is quite possible that investments will prove ineffective in raising participation in sport and physical activity. Indeed, it is often quoted that no previous Games have been successful in this respect [7]. Several challenges must be overcome for the London Games to avoid falling into this category.

In the light of the current economic climate, a key challenge is the attainment of adequate funding to support a physical activity participation legacy. Low cardio-respiratory fitness (CRF) is probably the World's most important non communicable disease public health risk factor. When considered alongside other all cause mortality risk factors, low CRF is of greater importance than the combined risks of smoking, obesity and diabetes [8]. The financial burden of physical inactivity to the UK alone has been estimated to be in the region of £7 billion/year [3]. Maintaining the status quo is simply not sustainable, a point made by Wanless as long ago as 2002 [9]. The inevitable implication, given that there is no new resource, is to re-direct current resources. This is a significant challenge for health care commissioners, who naturally want evidence of a potential return of any investment before reallocating resource. Early results from the Be Active programme in Birmingham suggest that this is achievable [10].

The next challenge is to ensure resource is allocated appropriately. A key ambition of the previous Labour Government's legacy action plan was to get two million more people more active by 2012 [2]. The Coalition Government has since dropped the physical activity target and recent data from the Active People Survey (the chosen measure for this legacy outcome) suggest there is little chance of the sport goal being met; only 111,800 more adults (~11% of one million target) are participating in sport since 2007/8 [11]. So where have things gone wrong? Experts have suggested that this is probably due to an over-emphasis on the achievements and heroism of elite athletes to motivate and inspire (known as the 'demonstration effect') as well as the majority of investment going towards developing sport facilities, neither of which are likely to engage people [7,12]. In Sheffield, we are building on the idea that a health legacy needs to be leveraged through a unified and city-wide approach that supports evidence-based initiatives (e.g. Table 1) targeting specific outcomes and by promoting the 2012 Games as a significant national celebration that transcends sport and is relevant to local communities, with benefits of participation linked to community participation rather than health - in short, by leveraging a 'festival effect' [7]. Our models suggest that our proposed investments will ultimately be cost-saving (e.g. [13]). Time will tell.

Another major challenge is "how will we know if the Games have benefitted health?" Key issues here are those of "attribution" and "additionality". For the former, it has been suggested that evaluation should not depend on generic national surveys, such as the Active People Survey [11], because any changes cannot be attributed to London 2012 participation initiatives [14]. Furthermore, such surveys cannot provide evidence of "additionality", meaning they cannot show that London 2012 programmes have increased physical activity/health to levels greater than could have been achieved by investment in alternative interventions. A broader description of these issues is provided by Professor Mike Weed in a recent Editorial [14]. Here he recommends that evaluation should focus on directly attributable effects and that detailed alternative scenarios, outlining what would have been most likely to happen in the absence of London 2012 initiatives, must be modelled for comparison purposes [14].

## Conclusions

Delivering a broad and sustained legacy of physical activity and health from the London 2012 Games is an ambitious target, especially since there is no evidence that previous Games have been successful in this respect. Thus, it is perhaps unsurprising that increases in physical activity participation in the lead up to London 2012 have been glacial at best. Although it is late in the day in terms of using the Olympic Games as a vehicle for increasing the health of UK citizens, opportunities still exist and in particular through the development of the NCSEM. Although the specific work programmes of each of the three NCSEM partner locations are still being clarified, it is clear that the specialty of Sport and Exercise Medicine has the potential to establish itself as a key player in the promotion of physical activity in the UK. The importance of physical activity in preventing and treating many diseases and conditions is indisputable. The challenge now is for those who hold the purse strings to be convinced of the need to invest to save and for key stakeholders to make the best use of any resources that become available. To achieve this, robust evaluation of current and future programmes is required with a focus on cost-benefit and 'real-cash returns' for those responsible for commissioning healthcare.

## List of abbreviations

CRF: Cardio-respiratory fitness; NCSEM: National Centre for Sport and Exercise Medicine; SEM: Sport and Exercise Medicine.

## Competing interests

The authors declare that they have no competing interests.

## Authors' contributions

GAT, RJC and SHT all contributed to manuscript preparation and approved the final draft for submission.

## Authors' information

GAT is a BASES accredited sport and exercise scientist (physiology research) and a Senior Research Fellow in the Centre for Sport and Exercise Science, Sheffield Hallam University. RJC is a Reader in Physical Activity and Health in the Centre for Sport and Exercise Science, Sheffield Hallam University. He is a British Psychological Society chartered psychologist and is project lead for Sheffield's National Centre for Sport and Exercise Medicine. SHT is a Consultant in Rheumatology/Sport & Exercise Medicine at Sheffield Teaching Hospitals NHS Foundation Trust and Clinical Lead for Sheffield's National Centre for Sport & Exercise Medicine.

## Pre-publication history

The pre-publication history for this paper can be accessed here:

http://www.biomedcentral.com/1741-7015/10/74/prepub
